# How sure am I? How text genre and question type shape comprehension calibration in primary and secondary school students

**DOI:** 10.3389/fpsyg.2025.1668045

**Published:** 2026-01-28

**Authors:** Alessandra Zagato, Eleonora Pizzigallo, Gerardo Pellegrino, Agnese Capodieci, Barbara Carretti, Chiara Mirandola

**Affiliations:** 1 Department of Human Sciences, IUL Telematic University, Florence, Italy; 2 Department of General Psychology, University of Padua, Padua, Italy; 3 Department of Psychology, University Salesian Institute of Venice, IUSVE, Venice, Italy

**Keywords:** calibration, comprehension, confidence ratings, postdictive judgments, question type, text genre

## Abstract

**Background:**

Metacognitive skills in text comprehension are fundamental for students' learning, yet their development may differ depending on text genre (narrative vs. expository), question type (factual vs. inferential), and educational level. However, little is known about how these factors influence students' calibration of comprehension.

**Methods:**

This study examined postdictive metacognitive judgments through student's confidence ratings collected after completing two reading comprehension tasks administered to 407 primary and secondary school students. These confidence judgements were then used to compute three calibration indices: Absolute Accuracy Index, Bias Index, and Discrimination Index, which assess the distance between the predicted and actual performance and the ability to discriminate correct from incorrect answers.

**Results:**

Analyses revealed that both primary and secondary school students performed similarly on narrative texts. However, primary school students scored significantly lower than secondary school students and overestimated their performance on expository passages and inferential questions. This pattern suggests that metacognitive calibration becomes more accurate with increased exposure to complex text genres and scholastic experience.

**Conclusions:**

These findings highlight the influence of text genre and question type on metacognitive calibration and provide useful implications for educational practices aimed at fostering metacognitive skills—such as teaching genre-specific reading strategies and training students to reflect on their comprehension.

## 1 Introduction

Imagine some students just finishing a reading comprehension test at school. They have some time to check their work before the teacher asks them to hand it in, so they go through the multiple-choice questions again, wondering how sure they are about their answers. Does their actual performance correlate with their ability to judge their comprehension? Does confidence in one's own performance vary depending on the text genre and question type? In this common scenario, students are implicitly engaging in a critical cognitive skill: the ability to accurately judge the understanding of what they have read—a key aspect of comprehension calibration, which can be seen as the relation between confidence and actual performance. The present study was aimed to shed light on this specific issue.

Despite extensive research on text comprehension, less attention has been paid to how students monitor and calibrate their understanding across different text genres and question types, especially across educational levels. This gap limits our understanding of how metacognitive accuracy develops during schooling. Primary and secondary school years are critical developmental periods for both comprehension and metacognitive skills (Roebers et al., 2009; de Bruin et al., 2011; Prinz et al., 2020). During these years, students progressively move from learning to read toward reading to learn, and they are increasingly required to understand, integrate, and evaluate information from texts to support learning. At the same time, they begin to develop and refine the metacognitive strategies needed to monitor their comprehension and regulate their learning processes. Understanding these aspects is therefore crucial for enhancing students' ability to calibrate their comprehension, improving teaching strategies, or providing support for struggling readers.

In the current study, we deepen the role of educational level, text genre, and comprehension level on calibration. Namely, we compare text comprehension calibration for narrative and expository texts and factual and inferential questions between primary and secondary-school students. In this framework, calibration is conceived as a specific outcome of metacognitive monitoring, as it reflects how closely students' confidence judgments align with their actual performance and provides the basis for subsequent control decisions, such as revising answers (Stone, 2000).

### 1.1 Text comprehension

The ability to comprehend a written text is the result of the construction of a coherent mental representation based on the integration of information from the text and prior knowledge (Kintsch, 1988, 1998; van Dijk and Kintsch, 1983). For example, the Construction–Integration model (Kintsch, 1988, 1998) conceives reading comprehension as the result of two recursive processes: the construction process, in which the text is transformed into a network of propositions (a textbase), automatically activating related prior knowledge and the integration process, in which this network is refined through constraint satisfaction, strengthening relevant links and weakening irrelevant ones, thereby creating a coherent mental representation (a situation model) that merges textual meaning with background knowledge. In addition, extensive research findings highlighted the importance of multiple variables, such as vocabulary (e.g. Perfetti et al., 2005; Cain and Oakhill, 2006), working memory (e.g. Peng et al., 2018), emotional-motivational factors (e.g. Rouet et al., 2017), and executive functions (e.g. Cirino et al., 2017). Metacognition also contributes to text understanding (Guthrie and Wigfield, 1999; McNamara and Magliano, 2009), by supporting monitoring and control processes that allow readers to evaluate their comprehension, detect inconsistencies or gaps, and adapt their reading strategies. Furthermore, other factors related to text characteristics or the way comprehension is assessed play a role. In particular, text genre (e.g., narrative vs. expository) and question type (e.g., factual vs. inferential) may modulate the outcome of a text comprehension task and the related calibration (Best et al., 2008; Basaraba et al., 2013; Eason et al., 2012; Clinton et al., 2020; Prinz et al., 2020; Mar et al., 2021).

### 1.2 Metacognitive monitoring and calibration

Metacognitive abilities are involved in the text comprehension process. One of the key metacognitive abilities is monitoring which, according to Brown (1978), refers to the ability to monitor one's activities while performing a task. In the context of a text comprehension task, monitoring skills enable one to judge accurately one's comprehension and responses. Ineffective monitoring does not only hinder performance in the ongoing task but also influences subsequent reading behavior: individuals who fail to recognize that they have not completely understood the content of a passage are less inclined to re-read or take other steps to enhance their comprehension (Mirandola et al., 2018; Prinz et al., 2020). In addition, in line with the *poor comprehension theory* (Yang et al., 2023), when readers do not construct a sufficiently coherent mental representation of the text, they lack the internal feedback needed to accurately judge their level of understanding, which in turn leads to poor metacognitive monitoring.

Monitoring abilities are closely related to *calibration*, which refers to the accuracy with which readers evaluate their own understanding of a text (Lin and Zabrucky, 1998). Calibration is often conceptualized as a specific aspect of *metacomprehension accuracy* that captures the correspondence between one's confidence judgments and actual comprehension performance (Prinz et al., 2020). In educational contexts, calibration accuracy represents a fundamental component of self-regulated learning (Zimmerman, 2008): students who can accurately judge what they have or have not understood are more likely to adapt their study strategies, estimate time effectively, and seek help when needed (Efklides, 2011). Conversely, lower calibration accuracy can lead to ineffective learning, as students may prematurely terminate study or fail to revisit misunderstood material (Hacker et al., 2008; Rawson et al., 2011). Calibration abilities increase with educational level (Prinz et al., 2020). Mirandola et al. (2018) for example showed that primary school pupils were more overconfident—showing a lower accuracy—in judging their performance compared to secondary school students. In the same vein, Prinz et al. (2020) found that young adults are more accurate in assessing their comprehension level than both primary and secondary school students. According to Prinz et al. (2020), by developing more efficient cognitive skills—such as advanced decoding skills and greater working-memory capacity—older students can focus less on “how to read” and more on understanding the content, thereby enhancing a greater calibration of comprehension (Roebers et al., 2007; de Bruin et al., 2011; Ikeda and Kitagami, 2013).

### 1.3 Effects of text genre and question type on comprehension and calibration

As mentioned, among the factors that could influence the outcome of a text comprehension task, our study focuses on text characteristics, such as text genre.

It has been shown that narrative texts are more familiar to readers, in particular to the youngest ones (Best et al., 2006). Narrative texts have a recognizable structure that facilitates their comprehension, whereas the same cannot be said of expository texts. Some studies have indicated that narrative texts are recalled and comprehended more readily than expository texts (e.g. Best et al., 2008; Zabrucky and Moore, 1999). Nonetheless, other studies found that expository texts are easier to understand and retrieve than narrative ones (Diakidoy, 2014; Saadatnia et al., 2017; Wolfe and Woodwyk, 2010), and some studies reported no difference between the two (Cunningham and Gall, 1990; Kintsch and Young, 1984; Roller and Schreiner, 1985; Prinz et al., 2020; Wannagat et al., 2022).

To clarify these conflicting results, Clinton et al. (2020) conducted a meta-analysis focusing on inferential comprehension. Their findings indicated an advantage in the narrative performance over expository texts. However, the age of the reader did not act as a moderator between text genre and inferential reading comprehension, contrary to previous findings. This discrepancy could stem from limited research controlling for various text genres and reader ages. Clinton et al. (2020) suggest that as readers mature, they may develop a heightened sensitivity to text and coherence standards across genres compared to children, potentially resulting in improved comprehension and performance monitoring. This advantage was confirmed by a more recent meta-analysis (Mar et al., 2021) that took a broader perspective, considering both memory and comprehension of text-based material: performance was about half a standard deviation better for narrative than for expository texts, in both cases, and the difference was greater in children and adolescents than in adults.

Other studies examined how text genre affects metacognition. In particular, the meta-analysis by Prinz et al. (2020) focused the attention on relative metacomprehension accuracy, that is how accurately learners can discriminate their comprehension of texts. Their results suggest that metacomprehension accuracy does not vary depending on text genre. The author noticed however that few studies analyzed narrative text, and most considered older students (from college onward). Furthermore, according to most reading comprehension models, readers can achieve varying degrees of comprehension (see the review by McNamara and Magliano, 2009). The Construction-Integration model by Kintsch (1998) and van Dijk and Kintsch (1983) states that, depending on the text and reader's characteristics, comprehension can occur at different representational levels. At a surface level, understanding involves explicit information. When information from adjacent sentences is combined, a text-base representation is formed. In contrast, when comprehension entails deeper processing—integrating different parts of a text with prior knowledge and elaborating through inferential reasoning—the reader constructs a situation-model representation. These distinctions are typically operationalized through different question types. Factual questions mainly tap text-based processing, requiring readers to retrieve explicit information from the text, whereas inferential questions involve constructing meaning that goes beyond what is explicitly stated, relying on situation-model representations (van den Broek, 1994).

From a metacognitive perspective, question type may also affect calibration accuracy. Factual questions provide more direct cues for evaluating correctness, potentially supporting better alignment between confidence and accuracy, while inferential questions involve uncertainty and integration processes that may increase over- or under confidence.

Considering this, we may expect differences in comprehension and calibration based on question type but empirical findings are mixed. Analyzing expository texts, Best et al. (2008) reported that fourth-graders were more likely to have difficulty answering inferential questions as opposed to factual ones. The authors also found that children with higher levels of knowledge were likely to comprehend texts better, in particular, expository texts. This finding fosters the importance of background knowledge in comprehending expository texts (Snow, 2002).

A recent study (Steiner et al., 2020) explored monitoring and control abilities across different question types, employing both cross-sectional and longitudinal approaches. Children read expository texts and then completed tests with different formats of questions, providing confidence ratings to assess monitoring and deciding whether to withdraw their answers to assess control. The authors investigated whether factual or inferential questions could affect children's confidence judgments. No clear differences in monitoring emerged between detail and inferential questions, and results could be influenced by test format (open-ended vs. true-false questions). Children's mean confidence judgments were slightly lower for incorrect answers to open-ended detail questions compared to inferential ones. Regarding factual questions, monitoring accuracy was greater with open-ended than true-false formats, although the nature of true-false questions has biased confidence due to a fifty-fifty chance of answering correctly. According to Prinz et al.'s (2020) meta-analysis (2020), a common finding is that comprehension calibration is higher for factual than for inferential questions (see, for example, Griffin et al., 2019, and Jaeger and Wiley, 2014). However, results from Prinz et al. (2020) are inconclusive, showing that this trend is not statistically significant. In contrast, Chen (2022) found that when inferential questions were used as criteria, readers' calibration was more accurate than when detailed questions were employed. These findings suggest the need for further research to clarify the role of question types in shaping calibration of comprehension.

Taken together, previous findings have shown mixed evidence regarding whether narrative or expository texts lead to better comprehension calibration, and similar ambiguity exists about the influence of factual vs. inferential questions on this type of task (see, for example, Prinz et al., 2020). Moreover, these findings provide a solid basis for expecting an interaction between text genre and question type, particularly across educational levels. Expository texts place higher demands on readers' background knowledge and present a less transparent causal organization in the text structure (e.g., Graesser et al., 1994; McKeown et al., 1992). For younger students, these characteristics may hinder the construction of a coherent situation model, making it more difficult to generate inferences required by inferential questions. In contrast, narrative passages rely on a more familiar and predictable structure (e.g., Mar et al., 2021), which can support bridging and elaborative inference making. As a result, inferential questions in expository texts may be especially challenging for primary school students—at both the comprehension and metacomprehension levels—where reduced coherence and lower activation of prior knowledge may limit the cues available for accurate calibration.

### 1.4 The present study

The present research aims to deepen our understanding of the role of text genre and question type in text comprehension calibration tasks in primary and secondary school students. Specifically, the study was guided by three main research questions: (1) Does calibration accuracy differ between primary and secondary school students? (2) Does calibration vary across text genres (narrative vs. expository) and question types (factual vs. inferential)?; and (3) Do these factors interact, reflecting developmental differences in familiarity with text types and metacognitive growth? To answer these questions, we therefore compared narrative and expository texts, as well as questions that involved retrieving details (factual questions) with questions demanding an elaboration of the content of the text (inferential questions). A multiple-choice test format was chosen for both types of questions, first because it enabled us to administer standardized reading comprehension tests, and second because it reflects the most typical situation in which students are tested at school.

After completing each test, students were asked to give a postdictive confidence judgment, which was used to compute three indices recommended by Schraw (2009): Absolute Accuracy Index (AAI), Bias Index (BI), and Discrimination Index (DI). These indices are widely used to capture distinct yet interrelated aspects of calibration: AAI provides an overall measure of how much the judgment of one's performance deviates from the actual performance. It refers to the discrepancy between the metacognitive assessment provided by the student and the actual performance; BI indicates whether the students tend to under- or over-estimate their performance. Finally, DI pertains to the ability to discriminate between correct and incorrect responses. Together, these indices emphasize the multidimensional nature of metacognitive accuracy (Schraw, 2009).

The aims of the present study were therefore twofold: (1) to replicate previous findings (e.g., Steiner et al., 2020; Mirandola et al., 2018) in typically developing children, further investigating the influence of confidence judgments on their reading comprehension and calibration during an educationally relevant task such as text comprehension; and (2) to examine whether students' calibration differs depending on text genre (narrative vs. expository) and question type (factual vs. inferential) across education levels.

According to previous findings (see Prinz et al., 2020; Mirandola et al., 2018) we expected that educational level would influence metacognitive monitoring and calibration of comprehension. In particular, we expected that primary school pupils would exhibit lower metacognitive abilities compared to secondary school students. However, it is still unclear whether such differences in comprehension calibration are solely due to individual factors (e.g., educational level) or also influenced by text characteristics (e.g., text genre and question type; see Prinz et al., 2020).

We therefore formulated the following hypotheses: (1) secondary school students would show higher calibration accuracy than primary school students; (2) calibration would vary across text genres and question types, such that primary school students were expected to show greater calibration accuracy for narrative texts, whereas secondary school students were expected to demonstrate better calibration accuracy for expository texts; (3) primary school students would exhibit lower calibration accuracy when evaluating inferential questions in expository texts compared to secondary school students and to narrative texts, whereas smaller differences between educational levels were expected for factual questions across both text genres.

## 2 Methods

### 2.1 Participants

A group of 470 Italian students attending primary school (4^th^ and 5^th^ grade) and secondary school (6^th^ and 7^th^ grade) in northern and central cities of Italy were enrolled in this study. Sixty-three participants were subsequently excluded from the analyses because they were diagnosed with learning disorders (*n* = 13) or difficulties (*n* = 22), or other neurodevelopmental disorders (*n* = 14), or because they had Italian as a second language (*n* = 14). These exclusion criteria were applied to ensure that differences in comprehension performance and calibration could be attributed to educational level and task characteristics rather than to language proficiency or specific learning impairments that might affect reading comprehension or metacognitive calibration. The final sample thus included 407 participants: 249 primary school pupils (132 females; *M*_age_ = 9.4 years; *SD*_age_ = 0.59) and 158 secondary school students (78 females). It should be noted that for secondary school students age was not reported due to ethical concerns.

Primary-school pupils were considered as a single group within the sample, as were secondary-school students, due to their similar instructional levels according to the Italian educational system.

The study was approved by the local ethical committee at the University of Padua. Recruitment occurred voluntarily, with written informed consent obtained from the students' parents before experimenting.

### 2.2 Materials

#### 2.2.1 Reading comprehension tasks

Two grade-appropriate texts taken from a standardized battery of reading comprehension tasks (Cornoldi and Carretti, 2016) were administered collectively to the students in their classroom (each class included 20 students on average). The readability of each text was assessed using the Gulpease Index (see Dell'Orletta et al., 2011). The resulting scores indicated that the texts—although all different from class to class—were appropriate for both primary and secondary school students and exhibited comparable levels of readability (see Tonelli et al., 2012). The specific Gulpease indexes are reported in Supplementary Table S1. Students were given one text and one answer sheet at a time. For each grade, one text was expository and the other one was narrative. For each text, there were 12 multiple-choice questions with 4 possible answers (only one of the four answers was correct). Six of the questions were designed to tap text-based-level comprehension, and the other six to tap inferential-level comprehension. An example of a text-based comprehension item is as follows: “Orlando saves his master because he manages to… (A) Hold him perfectly between the two rails; (B) Move him away from the rails; (C) Put him back on the track; (D) Stop the train”, whereas an example of an inferential question is: “Why is the title chosen for this passage not very appropriate? (A) Because it does not refer to the accident; (B) Because the protagonist is actually the elderly man; (C) Because it is too generic; (D) Because the dog's name is not mentioned”.

#### 2.2.2 Metacognitive calibration

After answering each comprehension question, students were asked to provide a postdictive judgment of confidence, evaluating their previous comprehension responses. This was an item-level retrospective confidence rating, which has been used in previous monitoring research (Mirandola et al., 2018; Tang et al., 2025). Students indicated (*a*) whether they believed their answer was correct or incorrect, and (*b*) their degree of confidence on a 5-point Likert scale (1 = *not sure at all*, 5 = *really sure*). The confidence rating was used to compute the metacognitive calibration indices (Absolute Accuracy Index, Bias Index, and Discrimination Index), as recommended by Schraw (2009).

### 2.3 Procedures

The assessment was conducted in one collective session during the students' class activities. The total time taken to complete the procedure was approximately 60 min.

After reading each text, first the narrative passage and then the expository one, students had to answer the 12 multiple-choice questions with no time limit. The order of texts and questions was consistent for all students, as we administered the original protocol of the test (Cornoldi and Carretti, 2016). Although fatigue effects were not formally assessed, students were invited to take a brief pause between the two tasks if needed. Students were allowed to re-read parts of the texts if they needed to, as indicated in the test manual. At the end of each comprehension test, students were asked to return the texts but keep their answer sheets. Then they were asked to complete the metacognitive calibration task by giving postdictive confidence judgments associated with their reading comprehension answers. This was done immediately after completing the comprehension task to limit forgetting of its content, as this could interfere with the metacomprehension task.

### 2.4 Data analysis

Comprehension performance was scored by the authors by assigning 1 point for each correct answer, and 0 for each incorrect answer (so the total comprehension score was the sum of the correct answers for each text). As already mentioned, two types of questions were included: factual questions (six for each type of text) regarding information that could be found in adjacent portions of the text; and inferential questions (six for each type of text) requiring inference and a more general understanding of the text. After summing all correct answers, we rescaled comprehension scores by converting them into *z*-scores within the whole sample. This transformation placed performances on a common metric across texts, question types, and educational levels while preserving relative differences between students. Positive *z*-scores indicate above-average performance and negative *z*-scores indicate below-average performance within the sample. These scores served as the dependent variables in our linear-mixed models of text comprehension performance.

Raw item-level postdictive confidence judgment scores were used to compute the metacognitive calibration indices (Schraw, 2009). The following metacognitive indices (Schraw, 2009) were calculated. (a) Absolute Accuracy Index (AAI), as a measure of the discrepancy between the confidence judgment of one's performance and the actual performance. The formula used is shown below:


$$\text{Absolute Accuracy Index }=\;\frac{1}{N}\;\sum_{i=1}^{N}{\left(c_{i}-\;p_{i}\right)}^{2}$$

*i* refers to the ith item within the calibration task for that student. Here, *c*_*i*_ is the confidence judgment for item *i* and *p*_*i*_ is the performance score on item *i*. The index ranges from 0 to 1, with higher values indicating lower calibration accuracy. (b) Bias Index (BI), is a measure of the degree of metacognitive accuracy. The measure used is shown below:


$$\begin{array}{l} Bias\;Index\;=\;\frac{1}{N}\;\overset{N}{\underset{i=1}{\sum }}\left(c_{i}-\;p_{i}\right)\; \end{array}$$

where *c*_*i*_ is the confidence rating for item *i* and *p*_*i*_ the performance score on item *i*. Its values range from 1 to −1: positive values farther from 0 indicate overestimation, while negative values farther from 0 indicate underestimation; (c) Discrimination Index (DI), as a measure of discrimination ability between correct and incorrect answers. The formula used is shown below:


$$\begin{array}{l} Discrimination\;Index\;=\;\frac{1}{N}\;\left[\overset{N_{c}}{\underset{i=1}{\sum }}\left(c_{i\;correct}-\overset{N_{i}}{\underset{i=1}{\sum }}c_{i\;incorrect}\right)\right]\; \end{array}$$

where *N*_*c*_ and *N*_*i*_correspond respectively to the number of correct and incorrect answers, while *c*_*i correct*_ and *c*_*i incorrect*_ are the confidence ratings for correct and incorrect items (*i*). Scores ranged between –∞ and +∞, with positive values indicating greater confidence in correctly judged answers and negative values indicating greater confidence in incorrectly judged answers.

As suggested by Schraw (2009), the calculation of these indices requires that scores were on an ordinal or continuous scale from 1 to 100. To enhance the interpretability of our results, the confidence scores, initially obtained from a Likert Scale, were converted to an ordinal scale ranging from 0.2 to 1, where 0.2 is equal to “*not sure at all”* and 1 to “*really sure”*. These metacognitive indices were used as the dependent variables in our linear-mixed models of metacognitive calibration.

All statistical analyses were conducted using R (R Studio Team, 2023). Analyses and Figures were run using the following R packages: *lme4* (Bates et al., 2015), *lmerTest* (Kuznetsova et al., 2017), *effects* (Fox and Hong, 2010), *emmeans* (Lenth et al., 2024), and *ggplot2* (Wickham, 2016).

As a preliminary analysis, we examined whether students at the same educational level but in different grades exhibited comparable reading comprehension performances using a linear mixed-effects model with grade as the predictor and participants as a random intercept. Linear mixed-effects models were used because the study employed a mixed within- and between-subject design, with repeated observations for each participant across text genre and question type.

Concerning data analysis, for each dependent variable (reading comprehension performance, AAI, BI, and DI), we compared four linear mixed-effects models to identify the most optimal one:

- a null model, including only random intercept;
- a first model, including text genre (TG; narrative vs. expository), question type (QT; factual vs. inferential), and educational level (primary vs. secondary school) as additive predictors;
- a second model, including the two-factor interaction between text genre and question type, and educational level as additive predictors. The two-way interaction indicates that the effect of question type differs depending on the text genre;
- a final model adding the three-factor interaction between genre, question type, and educational level. The three-way interaction indicates that the effect of question type on performance (or calibration) differs across text genres and that this pattern further depends on educational level.

In all models, participants were included as a random effect. The four models were compared using Chi-square test (χ^2^), Akaike information criterion (AIC), and Bayesian information criterion (BIC). We selected the model that best fit the data according to these criteria and used this model for parameter estimation: if the Chi-square test is significant, it indicates that adding more parameters significantly improves model fit; for AIC and BIC, lower scores indicate a better model fit. Marginal *R*^2^ was calculated to estimate the variance explained by the model.

For parameter estimation, we applied contrast coding using sum contrasts to all categorical predictors. This approach, as discussed by Brehm and Alday (2022), facilitates the interpretation of main effects and interactions by comparing each level of the predictor variables to the overall mean. This was particularly appropriate for our design aimed at testing interactions among categorical factors. The main effects represent the average effect of each factor—question type, genre, or educational level—on the dependent variables (text comprehension and metacognitive indices) while controlling for the other factors. This provides a measure of the impact of that factor independently of the other factors in the model.

When the interaction was found significant, *post-hoc* comparisons using Tukey's HSD test (Abdi and Williams, 2010) were conducted to compare the scores. Differences were considered significant when *p* ≤ 0.002 (according to Bonferroni correction for number of comparisons). The effect size was estimated using Cohen's *d* (Cohen, 1988).

## 3 Results

### 3.1 Preliminary analysis

First, we ran a linear mixed-effects model followed by pairwise comparisons between grades to examine whether students in different grades but at the same educational level performed similarly in reading comprehension. No significant differences in reading comprehension scores were found. Specifically, there was no difference between grades 4 and 5 (β = 0.10, *t* = 1.12, *p* = 0.681), nor between grades 6 and 7 (β = −0.19, *t* = −1.76, *p* = 0.294). Since no differences were found between grades, the following models were run using aggregated data by educational level.

### 3.2 Descriptive

Descriptive statistics for all variables are presented in Table 1 for primary school and secondary schools. The presence of missing values on the independent measures is due to the comprehensive inclusion of all participants who completed at least one task during the evaluation within the overall study sample.

**Table 1:** Descriptive statistics for primary and secondary school.

**Measures** **Primary school**	***N***	**Mean**	***SD***	**Min**	**Max**
**Reading comprehension performance**					
NT-Factual	249	0.09	1	−3	2
NT-Inferential	249	0.13	1.01	−3	2
ET-Factual	249	−0.08	0.96	−2	2
ET-Inferential	249	−0.25	0.94	−2	2
**Absolute Accuracy Index (AAI)**					
NT-Factual	249	0.28	0.16	0	1
NT-Inferential	249	0.29	0.16	0	1
ET-Factual	249	0.31	0.17	0	1
ET-Inferential	248	0.33	0.17	0	1
**Bias Index (BI)**					
NT-Factual	245	0.15	0.24	−1	1
NT-Inferential	239	0.19	0.25	−1	1
ET-Factual	241	0.24	0.26	−1	1
ET-Inferential	244	0.23	0.28	0	1
**Discrimination Index (DI)**					
NT-Factual	249	0.62	0.22	0	1
NT-Inferential	249	0.62	0.22	0	1
ET-Factual	249	0.57	0.24	0	1
ET-Inferential	249	0.55	0.24	0	1
**Secondary school**					
**Reading comprehension performance**					
NT-Factual	158	−0.14	0.98	−3	2
NT-Inferential	158	−0.21	0.95	−3	2
ET-Factual	158	0.13	1.04	−2	2
ET-Inferential	158	0.39	0.97	−2	2
**Absolute Accuracy Index (AAI)**					
NT-Factual	158	0.28	0.15	0	1
NT-Inferential	158	0.31	0.14	0	1
ET-Factual	158	0.27	0.17	0	1
ET-Inferential	158	0.24	0.14	0	1
**Bias Index (BI)**					
NT-Factual	154	0.2	0.24	0	1
NT-Inferential	156	0.18	0.23	0	1
ET-Factual	153	0.18	0.27	−1	1
ET-Inferential	154	0.07	0.26	0	1
**Discrimination Index (DI)**					
NT-Factual	158	0.61	0.21	0	1
NT-Inferential	158	0.59	0.21	0	1
ET-Factual	158	0.65	0.23	0	1
ET-Inferential	158	0.69	0.23	0	1

NT, narrative text; ET, expository text; Inferential, inferential questions; Factual, factual questions.

### 3.3 Linear mixed-effects models

The model comparison results, for each dependent variable, are reported in Supplementary Table S2. For reading comprehension performance, the three-factor interaction model (QuestionType × TextType × EducationalLevel) was the best fit (AIC = 4,364.3; BIC = 4,418.3; χ^2^ = 86.46, *p* < 0.001) compared to the additive model (QuestionType + TextType + EducationalLevel; AIC = 4,442.8; BIC = 4,475.1; χ^2^ = 0.98). Moreover, the three-factor interaction model provided the best fit for the Absolute Accuracy Index (AIC = −1,507.5; BIC = −1,453.5; χ^2^ = 43.16, *p* < 0.001), the Bias Index (AIC = 20.0; BIC = 73.7; χ^2^ = 44.11, *p* < 0.001), and the Discrimination Index (AIC = −319.6; BIC = −265.6; χ^2^ = 47.59, *p* < 0.001), compared to the additive model (Absolute Accuracy Index: AIC = −1,469.8; BIC = −1,437.4; χ^2^ = 11.41; Bias Index: AIC = 64.1; BIC = 96.3; χ^2^ = 10.76; Discrimination Index: AIC = −279.5; BIC = −247.2; χ^2^ = 10.15).

Table 2 shows the outcomes of the models exhibiting the optimal fit for each dependent variable, including reading comprehension performance and three metacognitive indices.

**Table 2:** Coefficients and statistical significance of the linear-mixed regression models.

**Outcome**	**β**	**Standard Error**	***df***	***t*-value**	***p*-value**	**Marginal *R*^2^**	**Conditional *R*^2^**
**Reading comprehension performance**						0.04	0.36
Intercept	0.01	0.04	407	0.22	0.824		
Question[Inferential]	0.01	0.02	1,221	0.43	0.671		
Text[ET]	0.04	0.02	1,221	1.95	0.052		
EL[Primary]	−0.03	0.04	407	−0.99	0.321		
Question[Inferential] × Text[ET]	0.01	0.02	1,221	0.71	0.477		
Question[Inferential] × EL[Primary]	−0.04	0.02	1,221	−1.9	0.057		
Text[ET] × EL[Primary]	−0.18	0.02	1,221	−8.71	**<0.001**		
Question[Inferential] × Text[ET] × EL[Primary]	−0.06	0.02	1,221	−3.18	**0.002**		
**Absolute Accuracy Index**						0.03	0.3
Intercept	0.29	0.01	407.1	53.23	**<0.001**		
Question[Inferential]	0.004	0.003	1,220.3	1.24	0.212		
Text[ET]	−0.002	0.003	1,220.3	−0.51	0.608		
EL[Primary]	0.02	0.01	407.1	2.88	**0.004**		
Question[Inferential] × Text[ET]	−0.01	0.003	1,220.3	−2.05	**0.041**		
Question[Inferential] × EL[Primary]	0.005	0.003	1,220.3	1.0.3	0.153		
Text[ET] × EL[Primary]	0.02	0.003	1,220.3	6.1	**<0.001**		
Question[Inferential] × Text[ET] × EL[Primary]	0.01	0.003	407.1	2.18	**0.029**		
**Bias Index**						0.03	0.32
Intercept	0.18	0.01	406.4	19.98	**<0.001**		
Question[Inferential]	−0.01	0.01	1,187.7	−2.30	**0.021**		
Text[ET]	0.001	0.01	1,186.9	0.12	0.906		
EL[Primary]	0.02	0.01	406.4	2.59	**0.010**		
Question[Inferential] × Text[ET]	−0.02	0.01	1,188.7	−2.94	**0.003**		
Question[Inferential] × EL[Primary]	0.02	0.01	1,187.7	3.48	**0.001**		
Text[ET] × EL[Primary]	0.031	0.01	1,186.9	5.67	**<0.001**		
Question[Inferential] × Text[ET] × EL[Primary]	0.004	0.01	1,188.7	0.72	0.474		
**Discrimination Index**						0.03	0.24
Intercept	0.61	0.01	407	83.03	**<0.001**		
Question[Inferential]	−0.001	0.01	1,221	−0.15	0.877		
Text[ET]	0.002	0.01	1,221	0.37	0.715		
EL[Primary]	−0.02	0.01	407	−2.97	**0.003**		
Question[Inferential] × Text[ET]	0.01	0.01	1,221	1.10	0.270		
Question[Inferential] × EL[Primary]	−0.01	0.01	1,221	−1.30	0.192		
Text[ET] × EL[Primary]	−0.03	0.01	1,221	−6.53	**<0.001**		
Question[Inferential] × Text[ET] × EL[Primary]	−0.01	0.01	1,221	−2.03	**0.042**		

ET, expository text; Inferential, inferential questions; EL, educational level. Bold values indicate statistically significant effects.

*Post hoc* pairwise comparisons are reported in Supplementary Table S3. Effect sizes (Cohen's *d*) are reported below for *post hoc* comparisons following significant interaction effects.

#### 3.3.1 Reading comprehension performance

The three-way interaction between question type, text type, and educational level was statistically significant (β = −0.06, *t* = −3.18, *p* = 0.002). The scores difference for the questions and text type changed depending on the educational level.

**Figure 1:**
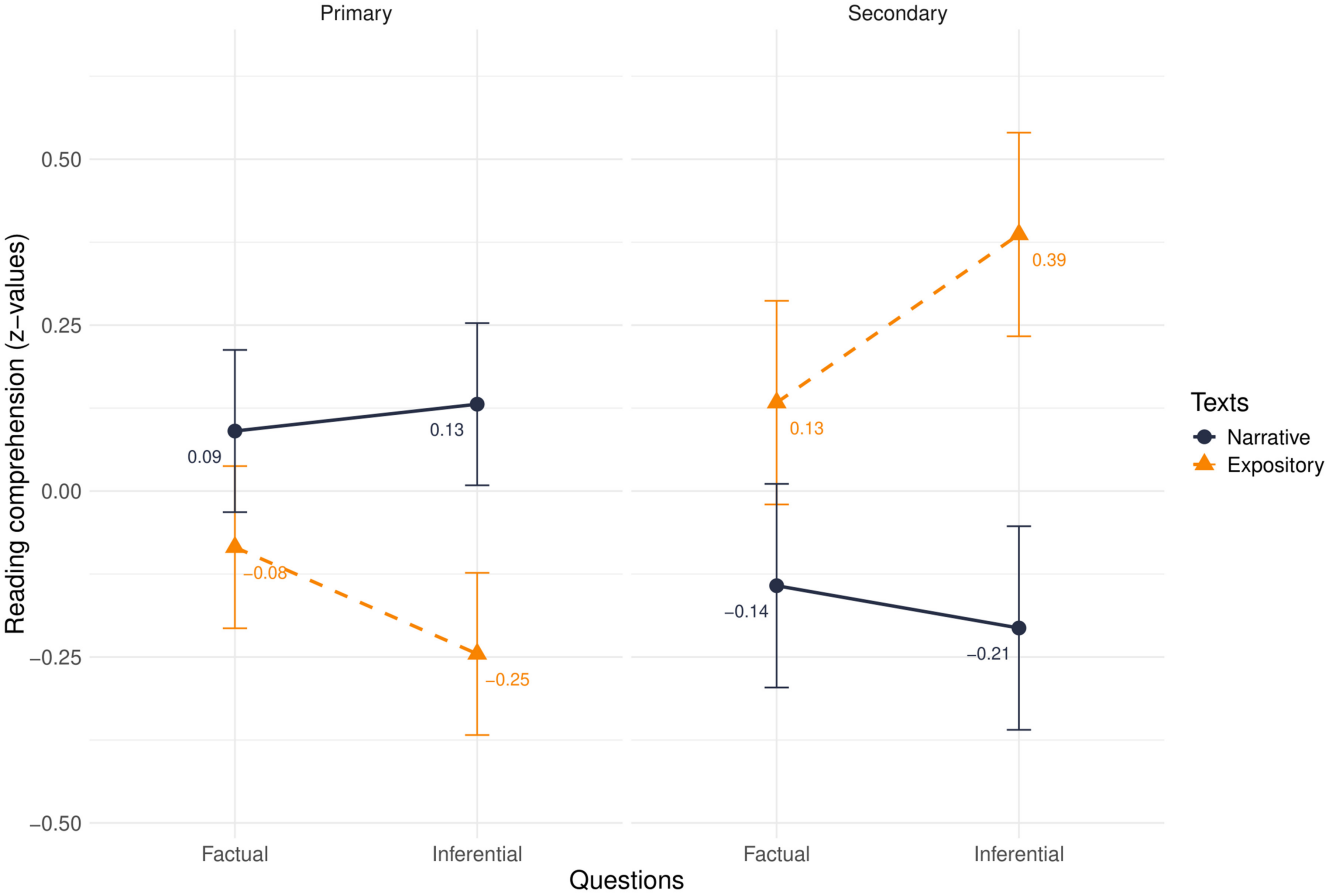
*Z*-scores in reading comprehension performance, divided by primary and secondary school in interaction with texts and questions.

Specifically, when reading narrative texts, there were no differences between primary and secondary school students for both factual and inferential questions. Regarding the expository text, there were no differences between educational levels for factual questions; however, for inferential questions, the performance of primary school students was lower than that of secondary school students (β = −0.63, *p* < 0.001; *d* = −0.79). The results of the analysis are reported in Table 2 and Figure 1.

#### 3.3.2 Absolute Accuracy Index

**Figure 2:**
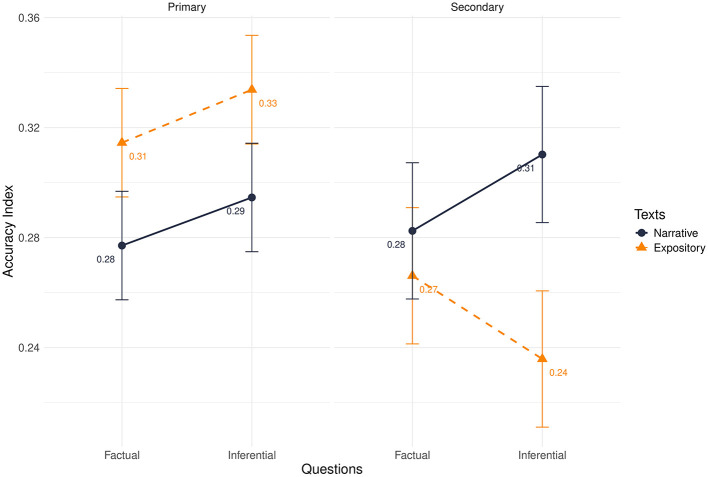
Absolute accuracy index divided by primary and secondary school in interaction with texts and questions. A higher score corresponds to a lower accuracy.

The effect of educational level was significant (β = 0.02, *t* = 2.88, *p* = 0.004), with primary school pupils being less accurate than secondary school students. The three-way interaction effect was significant (β = 0.01, *t* = 2.18, *p* = 0.03) and better clarified the main effect. The results of the analysis are synthesized in Table 2 and Figure 2.

Consistent with the performance results, for expository texts primary school students showed lower accuracy than secondary school students only when answering inferential questions (β = 0.10, *p* < 0.001; *d* = 0.72), with higher scores on the index corresponding to lower accuracy.

#### 3.3.3 Bias Index

**Figure 3:**
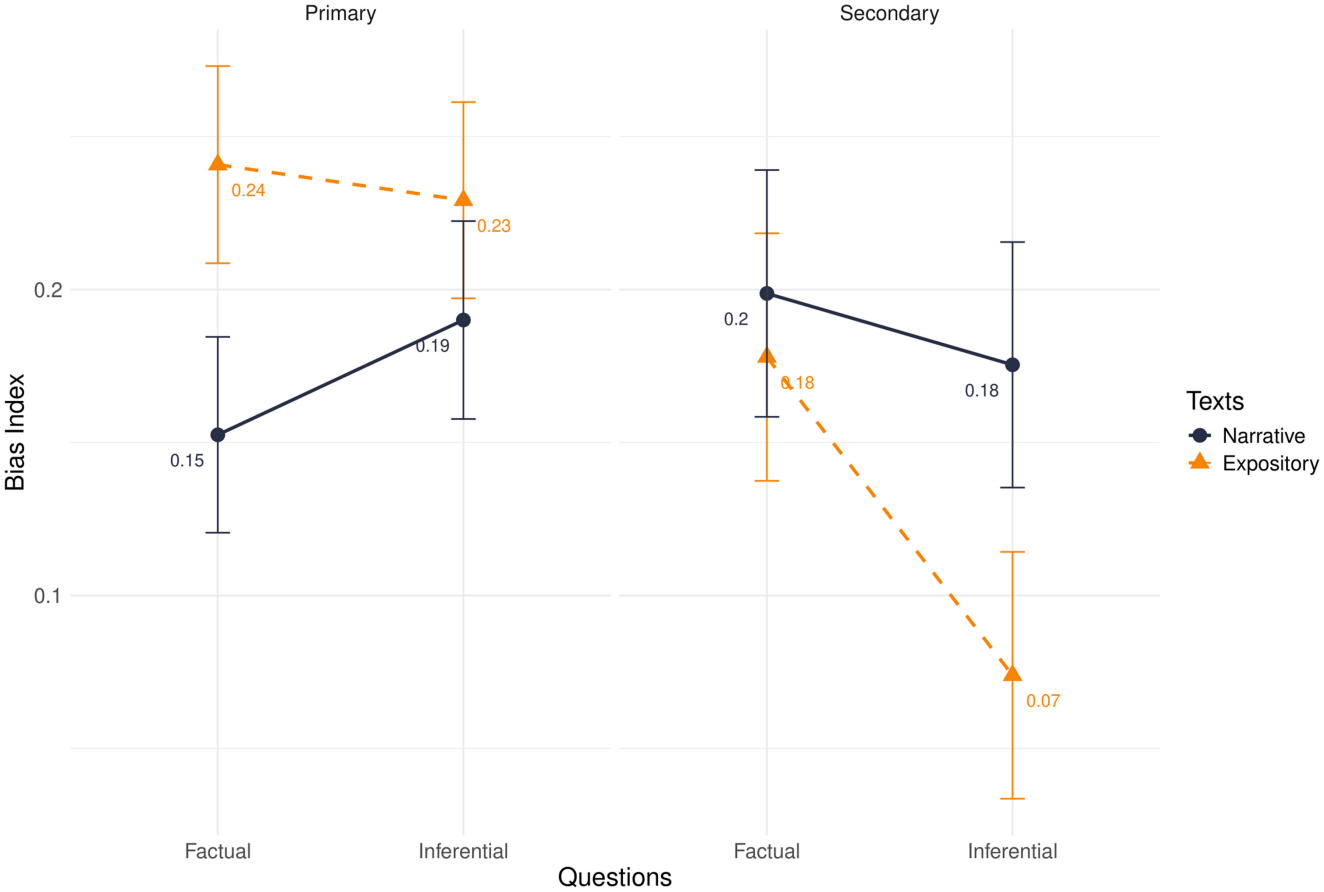
Bias index divided by primary and secondary school in interaction with texts and questions.

Regarding the bias index, the effects of question type (β = −0.01, *t* = −2.30, *p* = 0.021) and educational level (β = 0.02, *t* = 2.59, *p* = 0.010) were significant. There was no three-way interaction effect (Table 2, Figure 3); however, there were significant interactions between question type and educational level, and between text genre and educational level, aiding in the main effects interpretation.

In particular, primary school students showed a higher bias for expository texts (β = 0.03, *t* = 5.66, *p* < 0.001) compared to secondary school students. Additionally, they demonstrated a greater bias in inferential questions (β = 0.02, *t* = 3.48, *p* = 0.001).

#### 3.3.4 Discrimination Index

**Figure 4:**
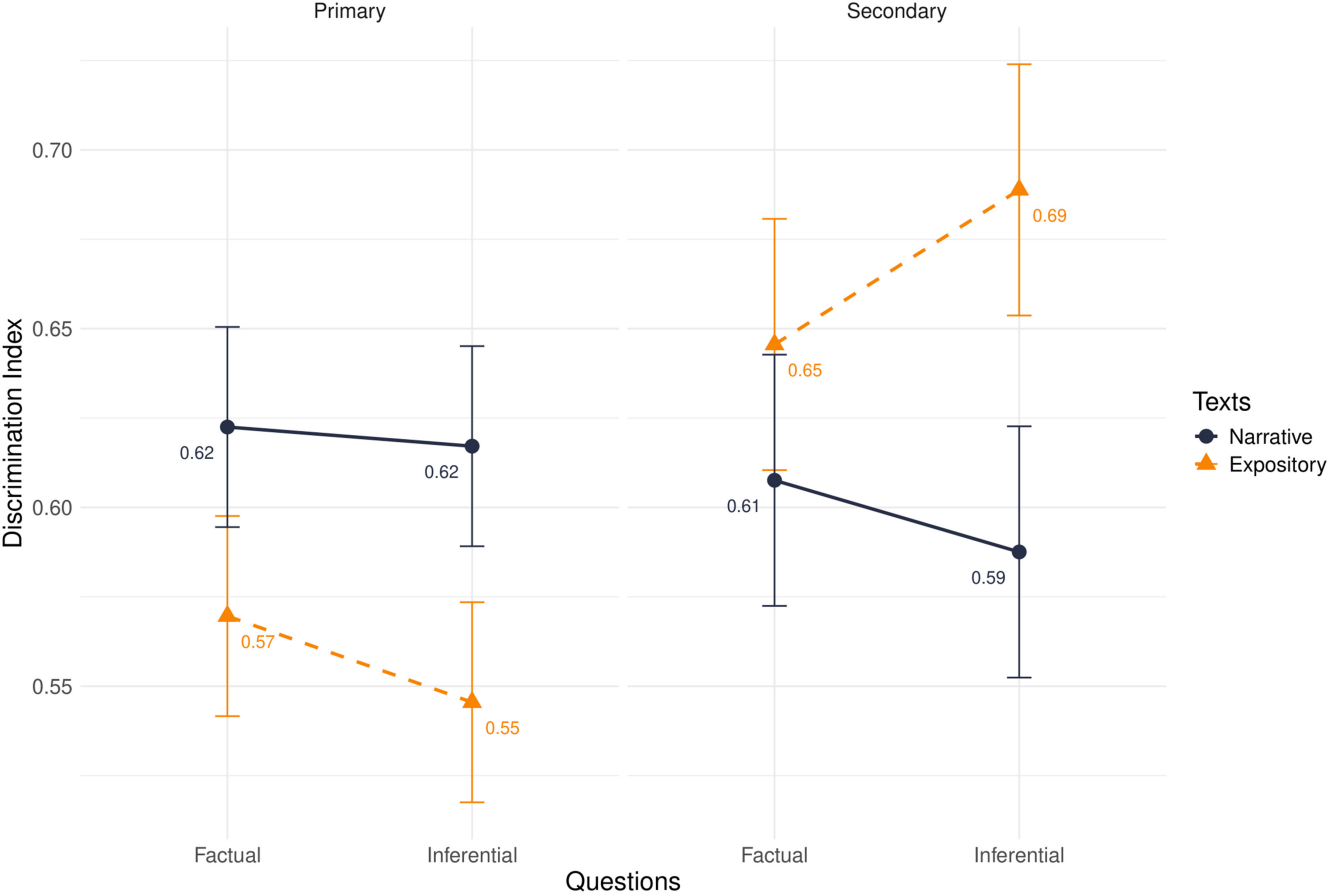
Discrimination index divided by primary and secondary school in interaction with texts and questions.

Finally, the discrimination index showed a significant effect of educational level (β = −0.02, *t* = −2.98, *p* = 0.003), in which primary school pupils were less confident about their correct answers than secondary school students. There also was a significant three-way interaction effect (β = −0.01, *t* = 2.03, *p* = 0.04) that clarified the main effect. The results of the analysis are summarized in Table 2 and Figure 4.

All students were confident about their correct answers (positive scores), but primary school students showed lower metacognitive awareness of their correct performance on the expository text when answering inferential questions compared to secondary school students (β = −0.14, *p* < 0.001; *d* = −0.72).

## 4 Discussion

The present study investigated whether text genre (i.e. narrative vs expository) and question type (i.e. factual vs inferential) differently affect typically developing students' calibration of comprehension at different educational levels. By focusing on how students judged the accuracy of their performance after completing two text comprehension tasks, we aimed to deepen our understanding of the factors contributing to effective metacognitive monitoring in educational relevant tasks. Overall, our findings revealed that both text genre and question type played a role in shaping text comprehension performance and its calibration.

Specifically, in our study, regarding narrative texts, there were no differences between educational levels, for both factual and inferential questions. However, when reading expository texts, primary school students scored significantly lower than secondary school students, particularly on inferential questions. This result, in line with recent reports (e.g. Mar et al., 2021), may reflect the intrinsic characteristics of texts and students' familiarity with them. These results partly align also with broader international studies (e.g. PIRLS, Palmerio and Emiletti, 2024) that highlight how expository digital texts tend to be more demanding than narrative passages in fourth-graders, although this trend is less pronounced in the Italian sample. As noted, both young students (Best et al., 2008; Williams et al., 2004) and adults (Wolfe and Woodwyk, 2010) tend to find narratives easier to understand than expository texts, probably due to greater exposure or familiarity (Duke, 2005), less varied and simpler text structure of narratives, and lower demand on prior knowledge of a given topic (e.g., McKeown et al., 1992; McNamara and Kintsch, 1996; Wolfe and Mienko, 2007). Indeed, narrative structures enable the reader to identify the main characters and key events and, by requiring less prior knowledge, facilitate the creation of a coherent thematic and causal representation of the text for a deep level of comprehension (Best et al., 2008; Graesser and Wiemer-Hastings, 1999). In contrast, understanding expository texts typically demands students to create a more complex, coherent representation that integrates the causal structure of the text and relevant prior knowledge (Brewer, 1980; Graesser et al., 2002, 1994; Trabasso and Magliano, 1996). Difficulty in constructing this representation can hinder deep comprehension and may affect students' performance on inferential questions.

Our results confirm that narrative texts are generally easy to understand, both at a surface and text-based representation level, for primary and secondary school students. In contrast, expository texts prove to be more challenging to comprehend at a deeper level, particularly for primary school pupils compared to secondary school students.

Further analyses revealed that the effects of text genre and question type also extended to the calibration of comprehension. We examined the interaction between the three metacognitive indices—Absolute Accuracy Index, Bias Index, and Discrimination Index (Schraw, 2009)—with text genre, question type, and educational level. No significant differences were observed between the two educational levels in the estimation of performance on narrative texts. In contrast, Absolute Accuracy Index values revealed that primary school pupils, compared to secondary school students, showed a lower accuracy in assessing their performance on expository texts, particularly when judging their responses to inferential questions.

More specifically, Bias Index values indicated that primary school pupils tended to overestimate their performance, regardless of text genre, on inferential questions and the expository text, compared to secondary school students. These results may reflect a challenge in accurately estimating their own performance on the more difficult-to-comprehend expository text and on the questions that require a deeper elaboration of the text. This could be due to their not fully developed ability to monitor their comprehension when facing more difficult tasks.

Text genre and question type also influenced the ability to discriminate between correct and incorrect answers among primary school pupils compared to secondary school students: it was only after reading narrative texts that pupils scored higher on the Discrimination Index. In other words, they were more accurate in judging the correctness/incorrectness of their answers when assessing their understanding of the narrative text. In contrast, for the more difficult-to-comprehend expository text, they were less confident about their responses to inferential questions. This suggests that both text characteristics and question type influenced their degree of confidence in the correctness of their answers. These results are consistent with the *poor comprehension theory* proposed by Yang et al. (2023), which suggests that poor comprehension offers few valid cues to inform judgments, thereby leading to low metacomprehension accuracy. In conclusion, the development of metacognitive abilities is also influenced by the educational level the students achieved. Our findings support the idea that as students' progress in their school careers they develop more effective metacognitive skills. This is because they are exposed to increasingly complex texts to study and academic demands which contribute to the improvement in their ability to monitor and estimate their performance.

Overall, our results suggest that primary school students' ability to assess their level of performance in test situations has not reached its full potential by 9 to 10 years old, especially when they are asked to judge their performance in cognitively demanding tasks. In contrast, secondary school students showed more effective calibration of their comprehension, demonstrating that, when facing complex tasks—such as reading and deeply understanding texts to study that are part of their daily school demands—they have developed higher metacognitive skills.

This is consistent with previous studies showing that metacognitive processes were not yet fully developed even in 9- to 11-year-olds (Roebers et al., 2009; de Bruin et al., 2011; García et al., 2016) and positively correlated with age and grade (Destan et al., 2017).

To sum up, our study suggests that both text genre and question type influence calibration in text comprehension tasks and that the transition from primary to secondary school is marked by an improvement in the efficiency of this metacognitive process.

The present study offers interesting findings, but it also has some limitations. For example, the order of text presentation was not counterbalanced because we followed the standardized protocol of the reading comprehension test; however, this may have introduced order effects. Moreover, the materials could have been the same for all participants, at least for those in the same school grade. The choice of texts was motivated by the necessity of employing standardized tests to facilitate the comparison of participants' text comprehension skills and their monitoring abilities across educational levels. Future research could benefit from employing more robust analytical approaches to address differences in item difficulty within and across tasks. Item Response Theory, for instance, allows for the evaluation of item properties and helps control for variability in item difficulty (e.g., Kulesz et al., 2016). Another complementary strategy would be to design reading comprehension batteries that combine common texts and items used across grade levels with grade-specific materials, thereby reducing item- and text-dependency in the resulting scores (Rodrigues et al., 2020).

Additionally, exposure to different types of texts may depend on each country's particular educational system. Therefore, further cross-cultural research should investigate whether the results observed in this study would be replicated or if any differences would emerge.

Another limitation concerns the postdictive judgments of confidence used to calculate the metacognitive indices (Schraw, 2009). Students rated how sure they were that their answer was correct on a five-point scale (1 = not sure at all, 5 = really sure), which we converted to a 0.2–1 ordinal scale for index computation. Although this format allowed us to compute the Absolute Accuracy Index, the Bias Index, and the Discrimination Index, it captured only degrees of confidence in correctness. This scale did not allow students to indicate that they believed they had answered a question—or all questions—incorrectly, nor to express complete uncertainty. As a result, calibration scores may have been biased toward overconfidence. Future studies may consider using a scale that captures the full range of perceived accuracy, allowing participants to judge a response as completely wrong—as well as completely correct.

Future research should also consider other individuals' variables that can contribute to understanding differences in calibration as the role of working memory capacity, which proved to be significant showing that lower capacity is associated with poorer metacomprehension accuracy (Griffin et al., 2008; Ikeda and Kitagami, 2013; Prinz et al., 2020) or motivation which can affect the willingness to review their own performance (e.g. Rouet et al., 2017). Furthermore, combining confidence judgments with other procedures, such as eye-tracking or think-aloud protocols, could provide more precise insights into real-time monitoring processes.

### 4.1 Implications for practice

Our findings highlight several practical implications for supporting metacomprehension at school. Younger students showed particular difficulty monitoring their understanding of expository texts and inferential questions, suggesting that teachers may need to provide more explicit scaffolding during these tasks. Strategies such as modeling how to identify text structure, activating relevant prior knowledge, and guiding students in generating bridging inferences may strengthen both comprehension and calibration accuracy.

From an educational point of view, calibration difficulties with expository texts underscore the importance of exposing children to different types of text from an early age. Their better performance with narrative texts has been attributed to cultural and educational factors that influence an individual's reading comprehension skills, thus it would be useful to introduce them to other text formats early on. This can be done by taking advantage of the close link between listening and reading comprehension, as numerous studies have demonstrated that listening comprehension is a good predictor of reading comprehension abilities (e.g. Lervåg et al., 2018). Additionally, several studies showed that intervention programs focusing on oral language abilities (including listening comprehension activities involving narratives) could have positive effects on reading comprehension skills (e.g., Silverman et al., 2020 for a meta-analysis) in both typical (e.g., Capodieci et al., 2021; Carretti et al., 2014) and atypical populations (Clarke et al., 2010). Educational programs that encourage students to increase their familiarity with expository texts will probably foster not only their text comprehension but also their metacognitive monitoring abilities.

Finally, calibration differences depending on text genre and question type underline the importance of promoting the development of a metacognitive and flexible approach to different comprehension tasks. Students should be able to analyze the task and select the most functional set of strategies for learning. For example, if a reading task requires only getting an idea of the topic, students should know that it would not be useful to read and reread the text and underline important pieces of information, just as it would not be to read a text, or a paragraph only once and immediately start outlining the central information. Furthermore, it is important to promote the adoption of a metacognitive approach during reading and study. At the end of each comprehension task, it could be beneficial to provide a “monitoring sheet” including some short questions encouraging students to assess their comprehension level, the text's difficulty and review specific passages when necessary.

Comprehension calibration should be promoted through specific programs to train students to become more aware of their comprehension during and after reading and learning to assess and control their performance. Of importance, teachers should be also trained to provide the best teaching and educational strategies developed to boost their students' metacognitive skills, such as encouraging them to reflect upon their control abilities instead of mainly focusing on the content of a lesson. Higher attention to both teachers' and students' abilities to share and implement metacognition might be an advisable tool for improvement.

## Data Availability

The datasets presented in this article are not readily available because the datasets generated and analyzed for this study are not publicly available due to copyright restrictions on the reading comprehension materials used in the assessment. Requests to access the datasets should be directed to eleonora.pizzigallo@phd.unipd.it.
