# Supplementary material for: How sure am I? How text genre and question type shape comprehension calibration in primary and secondary school students
Source: Front Psychol. 2026 Jan 28;16:1668045. doi: 10.3389/fpsyg.2025.1668045 (PMC12892345; doi:10.3389/fpsyg.2025.1668045)
Supplement: Supplementary file 1 [file Table_1.docx]

How sure am I? How text genre and question type shape comprehension calibration in primary and secondary school students

Supplementary Material

| **Table S1.** Gulpease Readability Indexes for each comprehension text | | | |
| --- | --- | --- | --- |
| **Text** | **Text Genre** | **Grade** | **Gulpease Index** |
| Il ponte dei bambini | N | 4 | 61.7% |
| La scoperta della patata | E | 4 | 55.2% |
| Storia di un cane | N | 5 | 54.0% |
| Tende, cavalli e libertà | E | 5 | 53.1% |
| Salvataggio della nave in Antartide | N | 6 | 49% |
| Il lago Aral | E | 6 | 59.6% |
| Il violino rubato | N | 7 | 58.1% |
| Le scimmie dell’isola di Koshima | E | 7 | 46.8% |
| *Note. N: narrative; E: expository.* |  |  |  |

| **Table S2.** Model comparison | | | | | | | | | |
| --- | --- | --- | --- | --- | --- | --- | --- | --- | --- |
|  | **Models** | **npar** | **AIC** | **BIC** | **logLik** | **deviance** | **χ²** | **Df** | **p-value** |
| **Reading Comprehension Performance** | | | |  |  |  |  |  |  |
|  | mod0 | 3 | 4437.8 | 4453.9 | -2215.9 | 4431.8 |  |  |  |
|  | mod1 | 6 | 4442.8 | 4475.1 | -2215.4 | 4430.8 | .98 | 3 | .805 |
|  | mod2 | 7 | 4444.8 | 4482.5 | -2215.4 | 4430.8 | .00 | 1 | 1 |
|  | mod3 | 10 | 4364.3 | 4418.3 | -2172.2 | 4344.3 | 86.46 | 3 | **<.001** |
| **Absolute Accuracy Index** | | |  |  |  |  |  |  |  |
|  | mod0 | 3 | -1464.4 | -1448.2 | 735.2 | -1470.4 |  |  |  |
|  | mod1 | 6 | -1469.8 | -1437.4 | 740.9 | -1481.8 | 11.41 | 3 | **.01** |
|  | mod2 | 7 | -1470.3 | -1432.5 | 742.2 | -1484.3 | 2.49 | 1 | .114 |
|  | mod3 | 10 | -1507.5 | -1453.5 | 763.7 | -1527.5 | 43.16 | 3 | **<.001** |
| **Bias Index** | |  |  |  |  |  |  |  |  |
|  | mod0 | 3 | 68.8 | 84.9 | -31.4 | 62.9 |  |  |  |
|  | mod1 | 6 | 64.1 | 96.3 | -26.0 | 52.1 | 10.76 | 3 | **.01** |
|  | mod2 | 7 | 58.1 | 95.7 | -22.1 | 44.1 | 7.95 | 1 | **.005** |
|  | mod3 | 10 | 20 | 73.7 | -.02 | .03 | 44.11 | 3 | **<.001** |
| **Discrimination Index** | | |  |  |  |  |  |  |  |
|  | mod0 | 3 | -275.4 | -259.2 | 140.7 | -281.4 |  |  |  |
|  | mod1 | 6 | -279.5 | -247.2 | 145.8 | -291.5 | 10.15 | 3 | .017 |
|  | mod2 | 7 | -277.9 | -240.2 | 145.9 | -291.9 | .43 | 1 | .513 |
|  | mod3 | 10 | -319.6 | -265.6 | 169.8 | -339.6 | 47.59 | 3 | **<.001** |
| ***Note.*** mod0 = null model; mod1 = QuestionType + TextType + EducationalLevel; mod2 = QuestionType X TextType + EducationalLevel; mod3 = QuestionType X TextType X EducationalLevel | | | | | | | | | |

| **Table S3.** Post hoc comparisons | | | | | | |
| --- | --- | --- | --- | --- | --- | --- |
| **Comparison** | **Estimate** | **SE** | **df** | **t.ratio** | **p.value** | **Effect**  **Size** |
| **Reading Comprehension Performance** |  |  |  |  |  |  |
| Inferential ET Primary - Factual ET Primary | -0.16 | 0.07 | 1.227 | -2.23 | 0.333 | -0.2 |
| Inferential ET Primary - Inferential NT Primary | -0.38 | 0.07 | 1.227 | -5.23 | **<.001** | -0.47 |
| Inferential ET Primary - Factual NT Primary | -0.34 | 0.07 | 1.227 | -4.66 | **<.001** | -0.42 |
| Inferential ET Primary - Inferential ET Secondary | -0.63 | 0.1 | 1.230 | -6.32 | **<.001** | -0.79 |
| Inferential ET Primary - Factual ET Secondary | -0.38 | 0.1 | 1.230 | -3.79 | 0.004 | -0.47 |
| Inferential ET Primary - Inferential NT Secondary | -0.04 | 0.1 | 1.230 | -0.39 | 1 | -0.05 |
| Inferential ET Primary - Factual NT Secondary | -0.1 | 0.1 | 1.230 | -1.03 | 0.97 | -0.13 |
| Factual ET Primary - Inferential NT Primary | -0.22 | 0.07 | 1.227 | -2.99 | 0.057 | -0.27 |
| Factual ET Primary - Factual NT Primary | -0.18 | 0.07 | 1.227 | -2.43 | 0.227 | -0.22 |
| Factual ET Primary - Inferential ET Secondary | -0.47 | 0.1 | 1.230 | -4.71 | **<.001** | -0.59 |
| Factual ET Primary - Factual ET Secondary | -0.22 | 0.1 | 1.230 | -2.18 | 0.365 | -0.27 |
| Factual ET Primary - Inferential NT Secondary | 0.12 | 0.1 | 1.230 | 1.22 | 0.927 | 0.15 |
| Factual ET Primary - Factual NT Secondary | 0.06 | 0.1 | 1.230 | 0.58 | 0.999 | 0.07 |
| Inferential NT Primary - Factual NT Primary | 0.04 | 0.07 | 1.227 | 0.56 | 0.999 | 0.05 |
| Inferential NT Primary - Inferential ET Secondary | -0.26 | 0.1 | 1.230 | -2.56 | 0.173 | -0.32 |
| Inferential NT Primary - Factual ET Secondary | 0 | 0.1 | 1.230 | -0.02 | 1 | 0 |
| Inferential NT Primary - Inferential NT Secondary | 0.34 | 0.1 | 1.230 | 3.37 | 0.017 | 0.42 |
| Inferential NT Primary - Factual NT Secondary | 0.27 | 0.1 | 1.230 | 2.74 | 0.113 | 0.34 |
| Factual NT Primary - Inferential ET Secondary | -0.3 | 0.1 | 1.230 | -2.96 | 0.062 | -0.37 |
| Factual NT Primary - Factual ET Secondary | -0.04 | 0.1 | 1.230 | -0.43 | 1 | -0.05 |
| Factual NT Primary - Inferential NT Secondary | 0.3 | 0.1 | 1.230 | 2.97 | 0.061 | 0.37 |
| Factual NT Primary - Factual NT Secondary | 0.23 | 0.1 | 1.230 | 2.33 | 0.277 | 0.29 |
| Inferential ET Secondary - Factual ET Secondary | 0.25 | 0.09 | 1.227 | 2.8 | 0.095 | 0.32 |
| Inferential ET Secondary - Inferential NT Secondary | 0.59 | 0.09 | 1.227 | 6.56 | **<.001** | 0.74 |
| Inferential ET Secondary - Factual NT Secondary | 0.53 | 0.09 | 1.227 | 5.85 | **<.001** | 0.66 |
| Factual ET Secondary - Inferential NT Secondary | 0.34 | 0.09 | 1.227 | 3.76 | 0.004 | 0.42 |
| Factual ET Secondary - Factual NT Secondary | 0.28 | 0.09 | 1.227 | 3.05 | 0.048 | 0.34 |
| Inferential NT Secondary - Factual NT Secondary | -0.06 | 0.09 | 1.227 | -0.71 | 0.997 | -0.08 |
| **Absolute Accuracy Index** |  |  |  |  |  |  |
| Inferential ET Primary - Factual ET Primary | 0.02 | 0.01 | 1.227 | 1.59 | 0.757 | 0.14 |
| Inferential ET Primary - Inferential NT Primary | 0.04 | 0.01 | 1.227 | 3.23 | 0.028 | 0.29 |
| Inferential ET Primary - Factual NT Primary | 0.06 | 0.01 | 1.227 | 4.67 | **<.001** | 0.42 |
| Inferential ET Primary - Inferential ET Secondary | 0.1 | 0.02 | 1.335 | 6.06 | **<.001** | 0.73 |
| Inferential ET Primary - Factual ET Secondary | 0.07 | 0.02 | 1.335 | 4.19 | **0.001** | 0.5 |
| Inferential ET Primary - Inferential NT Secondary | 0.02 | 0.02 | 1.335 | 1.46 | 0.829 | 0.17 |
| Inferential ET Primary - Factual NT Secondary | 0.05 | 0.02 | 1.335 | 3.18 | 0.033 | 0.38 |
| Factual ET Primary - Inferential NT Primary | 0.02 | 0.01 | 1.226 | 1.64 | 0.723 | 0.15 |
| Factual ET Primary - Factual NT Primary | 0.04 | 0.01 | 1.226 | 3.09 | 0.043 | 0.28 |
| Factual ET Primary - Inferential ET Secondary | 0.08 | 0.02 | 1.334 | 4.87 | **<.001** | 0.58 |
| Factual ET Primary - Factual ET Secondary | 0.05 | 0.02 | 1.334 | 3 | 0.056 | 0.36 |
| Factual ET Primary - Inferential NT Secondary | 0 | 0.02 | 1.334 | 0.27 | 1 | 0.03 |
| Factual ET Primary - Factual NT Secondary | 0.03 | 0.02 | 1.334 | 1.99 | 0.491 | 0.24 |
| Inferential NT Primary - Factual NT Primary | 0.02 | 0.01 | 1.226 | 1.44 | 0.837 | 0.13 |
| Inferential NT Primary - Inferential ET Secondary | 0.06 | 0.02 | 1.334 | 3.64 | 0.007 | 0.44 |
| Inferential NT Primary - Factual ET Secondary | 0.03 | 0.02 | 1.334 | 1.76 | 0.645 | 0.21 |
| Inferential NT Primary - Inferential NT Secondary | -0.02 | 0.02 | 1.334 | -0.97 | 0.979 | -0.12 |
| Inferential NT Primary - Factual NT Secondary | 0.01 | 0.02 | 1.334 | 0.75 | 0.995 | 0.09 |
| Factual NT Primary - Inferential ET Secondary | 0.04 | 0.02 | 1.334 | 2.56 | 0.174 | 0.31 |
| Factual NT Primary - Factual ET Secondary | 0.01 | 0.02 | 1.334 | 0.68 | 0.997 | 0.08 |
| Factual NT Primary - Inferential NT Secondary | -0.03 | 0.02 | 1.334 | -2.05 | 0.448 | -0.25 |
| Factual NT Primary - Factual NT Secondary | -0.01 | 0.02 | 1.334 | -0.33 | 1 | -0.04 |
| Inferential ET Secondary - Factual ET Secondary | -0.03 | 0.02 | 1.226 | -1.99 | 0.49 | -0.22 |
| Inferential ET Secondary - Inferential NT Secondary | -0.07 | 0.02 | 1.226 | -4.89 | **<.001** | -0.55 |
| Inferential ET Secondary - Factual NT Secondary | -0.05 | 0.02 | 1.226 | -3.06 | 0.046 | -0.35 |
| Factual ET Secondary - Inferential NT Secondary | -0.04 | 0.02 | 1.226 | -2.9 | 0.074 | -0.33 |
| Factual ET Secondary - Factual NT Secondary | -0.02 | 0.02 | 1.226 | -1.07 | 0.962 | -0.12 |
| Inferential NT Secondary - Factual NT Secondary | 0.03 | 0.02 | 1.226 | 1.83 | 0.603 | 0.21 |
| **Bias Index** |  |  |  |  |  |  |
| Inferential ET Primary - Factual ET Primary | -0.01 | 0.02 | 1.194 | -0.6 | 0.999 | -0.05 |
| Inferential ET Primary - Inferential NT Primary | 0.04 | 0.02 | 1.194 | 2.01 | 0.477 | 0.18 |
| Inferential ET Primary - Factual NT Primary | 0.08 | 0.02 | 1.192 | 3.96 | 0.002 | 0.36 |
| Inferential ET Primary - Inferential ET Secondary | 0.16 | 0.03 | 1.278 | 5.91 | **<.001** | 0.73 |
| Inferential ET Primary - Factual ET Secondary | 0.05 | 0.03 | 1.280 | 1.95 | 0.518 | 0.24 |
| Inferential ET Primary - Inferential NT Secondary | 0.05 | 0.03 | 1.272 | 2.05 | 0.445 | 0.25 |
| Inferential ET Primary - Factual NT Secondary | 0.03 | 0.03 | 1.278 | 1.16 | 0.943 | 0.14 |
| Factual ET Primary - Inferential NT Primary | 0.05 | 0.02 | 1.198 | 2.59 | 0.159 | 0.24 |
| Factual ET Primary - Factual NT Primary | 0.09 | 0.02 | 1.193 | 4.54 | **<.001** | 0.41 |
| Factual ET Primary - Inferential ET Secondary | 0.17 | 0.03 | 1.281 | 6.34 | **<.001** | 0.78 |
| Factual ET Primary - Factual ET Secondary | 0.06 | 0.03 | 1.284 | 2.39 | 0.249 | 0.29 |
| Factual ET Primary - Inferential NT Secondary | 0.07 | 0.03 | 1.275 | 2.49 | 0.199 | 0.31 |
| Factual ET Primary - Factual NT Secondary | 0.04 | 0.03 | 1.281 | 1.6 | 0.751 | 0.2 |
| Inferential NT Primary - Factual NT Primary | 0.04 | 0.02 | 1.195 | 1.92 | 0.534 | 0.18 |
| Inferential NT Primary - Inferential ET Secondary | 0.12 | 0.03 | 1.283 | 4.41 | **<.001** | 0.54 |
| Inferential NT Primary - Factual ET Secondary | 0.01 | 0.03 | 1.286 | 0.46 | 1 | 0.06 |
| Inferential NT Primary - Inferential NT Secondary | 0.01 | 0.03 | 1.278 | 0.56 | 0.999 | 0.07 |
| Inferential NT Primary - Factual NT Secondary | -0.01 | 0.03 | 1.283 | -0.33 | 1 | -0.04 |
| Factual NT Primary - Inferential ET Secondary | 0.08 | 0.03 | 1.277 | 2.99 | 0.056 | 0.37 |
| Factual NT Primary - Factual ET Secondary | -0.03 | 0.03 | 1.279 | -0.97 | 0.979 | -0.12 |
| Factual NT Primary - Inferential NT Secondary | -0.02 | 0.03 | 1.270 | -0.87 | 0.988 | -0.11 |
| Factual NT Primary - Factual NT Secondary | -0.05 | 0.03 | 1.277 | -1.76 | 0.647 | -0.22 |
| Inferential ET Secondary - Factual ET Secondary | -0.1 | 0.02 | 1.195 | -4.25 | **0.001** | -0.49 |
| Inferential ET Secondary - Inferential NT Secondary | -0.1 | 0.02 | 1.192 | -4.17 | **0.001** | -0.48 |
| Inferential ET Secondary - Factual NT Secondary | -0.12 | 0.02 | 1.194 | -5.11 | **<.001** | -0.59 |
| Factual ET Secondary - Inferential NT Secondary | 0 | 0.02 | 1.191 | 0.1 | 1 | 0.01 |
| Factual ET Secondary - Factual NT Secondary | -0.02 | 0.02 | 1.195 | -0.85 | 0.99 | -0.1 |
| Inferential NT Secondary - Factual NT Secondary | -0.02 | 0.02 | 1.192 | -0.96 | 0.98 | -0.11 |
| **Discrimination Index** |  |  |  |  |  |  |
| Inferential ET Primary - Factual ET Primary | -0.02 | 0.02 | 1.227 | -1.35 | 0.879 | -0.12 |
| Inferential ET Primary - Inferential NT Primary | -0.07 | 0.02 | 1.227 | -4.02 | 0.002 | -0.36 |
| Inferential ET Primary - Factual NT Primary | -0.08 | 0.02 | 12.270 | -4.32 | **<.001** | -0.39 |
| Inferential ET Primary - Inferential ET Secondary | -0.14 | 0.02 | 1.429 | -6.26 | **<.001** | -0.72 |
| Inferential ET Primary - Factual ET Secondary | -0.1 | 0.02 | 1.429 | -4.37 | **<.001** | -0.5 |
| Inferential ET Primary - Inferential NT Secondary | -0.04 | 0.02 | 1.429 | -1.84 | 0.596 | -0.21 |
| Inferential ET Primary - Factual NT Secondary | -0.06 | 0.02 | 1.429 | -2.71 | 0.12 | -0.31 |
| Factual ET Primary - Inferential NT Primary | -0.05 | 0.02 | 1.227 | -2.67 | 0.134 | -0.24 |
| Factual ET Primary - Factual NT Primary | -0.05 | 0.02 | 1.227 | -2.97 | 0.061 | -0.27 |
| Factual ET Primary - Inferential ET Secondary | -0.12 | 0.02 | 1.429 | -5.21 | **<.001** | -0.6 |
| Factual ET Primary - Factual ET Secondary | -0.08 | 0.02 | 1.429 | -3.32 | 0.021 | -0.38 |
| Factual ET Primary - Inferential NT Secondary | -0.02 | 0.02 | 1.429 | -0.78 | 0.994 | -0.09 |
| Factual ET Primary - Factual NT Secondary | -0.04 | 0.02 | 1.429 | -1.66 | 0.714 | -0.19 |
| Inferential NT Primary - Factual NT Primary | -0.01 | 0.02 | 1.227 | -0.3 | 1 | -0.03 |
| Inferential NT Primary - Inferential ET Secondary | -0.07 | 0.02 | 1.429 | -3.13 | 0.038 | -0.36 |
| Inferential NT Primary - Factual ET Secondary | -0.03 | 0.02 | 1.429 | -1.24 | 0.919 | -0.14 |
| Inferential NT Primary - Inferential NT Secondary | 0.03 | 0.02 | 1.429 | 1.29 | 0.902 | 0.15 |
| Inferential NT Primary - Factual NT Secondary | 0.01 | 0.02 | 1.429 | 0.42 | 1 | 0.05 |
| Factual NT Primary - Inferential ET Secondary | -0.07 | 0.02 | 1.429 | -2.9 | 0.074 | -0.33 |
| Factual NT Primary - Factual ET Secondary | -0.02 | 0.02 | 1.429 | -1.01 | 0.973 | -0.12 |
| Factual NT Primary - Inferential NT Secondary | 0.03 | 0.02 | 1.429 | 1.53 | 0.794 | 0.18 |
| Factual NT Primary - Factual NT Secondary | 0.01 | 0.02 | 1.429 | 0.65 | 0.998 | 0.08 |
| Inferential ET Secondary - Factual ET Secondary | 0.04 | 0.02 | 1.227 | 1.93 | 0.529 | 0.22 |
| Inferential ET Secondary - Inferential NT Secondary | 0.1 | 0.02 | 1.227 | 4.53 | **<.001** | 0.51 |
| Inferential ET Secondary - Factual NT Secondary | 0.08 | 0.02 | 1.227 | 3.63 | 0.007 | 0.41 |
| Factual ET Secondary - Inferential NT Secondary | 0.06 | 0.02 | 1.227 | 2.59 | 0.16 | 0.29 |
| Factual ET Secondary - Factual NT Secondary | 0.04 | 0.02 | 1.227 | 1.7 | 0.689 | 0.19 |
| Inferential NT Secondary - Factual NT Secondary | -0.02 | 0.02 | 1.227 | -0.9 | 0.986 | -0.1 |
| ***Note.*** NT: narrative text; ET: expository text; Inferential: inferential questions; Factual: factual questions | | | | | | |
